# Disruption of the *UPC2* Gene Enhances Fluconazole Antifungal Activity by Inhibiting *HAC1* mRNA Splicing in *Candida albicans*

**DOI:** 10.3390/pathogens15060629

**Published:** 2026-06-12

**Authors:** Jinhua Yu, Bingchen Jiang, Juan Xiong, Xiaojing Xu, Liping Xu, Yuanying Jiang, Hui Lu

**Affiliations:** 1 Department of Pharmacy, Shanghai Tenth People’s Hospital, School of Medicine, Tongji University, Shanghai 200331, China; 2 Shanghai Municipal Hospital of Traditional Chinese Medicine, Shanghai 200071, China; 3 Key Laboratory of Pathogen-Host Interaction, Ministry of Education, School of Medicine, Tongji University, Shanghai 200331, China

**Keywords:** Upc2, unfolded protein response, HAC1 mRNA splicing, *Candida albicans*

## Abstract

Azole resistance in *Candida albicans* is an increasing clinical challenge. Upc2 is a key transcription factor regulating ergosterol biosynthesis, but its additional roles in azole tolerance remain unclear. This study investigated whether Upc2 contributes to azole resistance through pathways beyond ergosterol synthesis. Chemical sensitivity screening, RNA sequencing, flow cytometry, and molecular assays were performed to compare wild-type *C. albicans* and the *upc2*Δ/*upc2*Δ mutant under fluconazole (FLC) treatment. The *UPC2* gene deletion affected physiological processes that are dependent on the calcineurin signaling pathway and led to an overall negative enrichment trend in the unfolded protein response (UPR) pathway gene set. Mechanistically, the *UPC2* gene deletion impaired unconventional splicing of *HAC1* mRNA, leading to accumulation of unfolded proteins and phenotypically its deletion enhanced sensitivity of *C. albicans* to FLC in planktonic growth, hyphal development, and biofilm formation. Our findings reveal that Upc2 regulates proteostasis in *C. albicans*, and its absence enhances FLC efficacy by disrupting the UPR pathway. Targeting Upc2-mediated UPR signaling may represent a promising strategy to combat azole resistance.

## 1. Introduction

*Candida albicans* is a notable human fungal pathogen capable of colonizing various anatomical sites, including the skin, mucous membranes, and gastrointestinal tract [1,2]. Disruptions in the host’s immune system or microbiota can precipitate both localized and systemic infections by *C. albicans*, posing a substantial risk to immunocompromised individuals due to the potential for life-threatening conditions [3]. Azoles represent the primary therapeutic option for candidiasis management. However, due to their fungistatic nature, *C. albicans* demonstrates an intrinsic tolerance to azoles, and extended exposure can facilitate the development of azole resistance [4]. Consequently, there is an imperative need to identify novel molecular targets and develop new antifungal agents to mitigate azole tolerance, counteract drug resistance in *C. albicans*, and thereby augment the efficacy of azole-based treatments [5].

Research has demonstrated that targeting ergosterol synthase enzymes other than Erg11 can further inhibit ergosterol biosynthesis, thereby significantly enhancing the antifungal efficacy of azoles [6]. Pitavastatin, a statin, has been shown to inhibit the activity of 3-hydroxy-3-methylglutaryl-CoA reductase (Hmg1), thereby enhancing the antifungal activity of fluconazole (FLC)against azole-resistant *Candida* species and even imparting fungicidal properties to FLC [7]. However, it should be noted that statins may cause adverse nonspecific effects and potential drug interactions [8]. Terbinafine, an inhibitor of Erg1, exhibits substantial antifungal efficacy against *Aspergillus* and azole-resistant *Candida* species when used in conjunction with azoles [9,10]. Nonetheless, the emergence of resistance to terbinafine among pathogenic fungi limits its clinical application [11]. The small molecule CZ66 (ZINC database no. 1772579137) has been shown to inhibit Erg251 activity, thereby impeding the synthesis of 14α-methylsterol and reducing the tolerance of *C. albicans* to azoles, while concurrently augmenting their antifungal efficacy [12]. However, CZ66 is characterized by a short half-life in murine models and does not significantly enhance the in vivo antifungal efficacy of azoles [12]. Consequently, there is an urgent need to develop novel strategies to inhibit ergosterol synthesis and potentiate the antifungal activity of azoles.

Upc2 serves as a pivotal transcription factor in the regulation of ergosterol biosynthesis. Structurally, Upc2 comprises a nuclear localization signal (NLS) at its N-terminus, a DNA-binding domain (DBD), a ligand-binding domain (LBD) at its C-terminus, and an activation loop [13]. Under conditions of normal intracellular ergosterol levels, ergosterol associates with the LBD, while Hsp90 interacts with the activation loop of Upc2, resulting in its retention within the cytoplasm [13,14]. Upon *C. albicans’* exposure to azoles, which cause a reduction in ergosterol levels, ergosterol dissociates from Upc2, inducing conformational changes in the C-terminal activation loop. This conformational change facilitates the dissociation of Hsp90 from Upc2. Subsequently, the nuclear importin Srp1 binds to the NLS of the now free Upc2, mediating its transport into the nucleus. Once inside the nucleus, the DBD of Upc2 binds to sterol response elements (SREs) located in the promoter regions, thereby activating the transcription of ergosterol biosynthesis genes (*ERGs*) [14].

The transcription factor Upc2 is pivotal in mediating resistance and tolerance to azoles in *Candida* species. Tan et al. demonstrated that Upc2 is capable of sensing intracellular ergosterol levels in fungi, subsequently regulating both the biosynthesis and uptake of ergosterol, which contributes to azole resistance in *Candida* species [14]. Vasicek et al. reported that activation of Upc2 induces azole resistance in *C. albicans*, whereas dysfunction of Upc2 enhances the sensitivity of *C. albicans* to azoles [15]. Wang et al. conducted an analysis of the transcriptional level of the *UPC2* gene in 319 clinical strains of *Candida tropicalis*, revealing a linear positive correlation between the gene’s transcriptional level and FLC resistance in *Candida tropicalis* [16]. Li et al. identified that Upc2 is integral to azole resistance in *Candida auris* by facilitating ergosterol biosynthesis and upregulating the expression of the drug transporter Mdr1 [17]. Furthermore, Vu et al. demonstrated that the transcription factor Upc2A in *Candida glabrata* can directly bind to the promoter region of the *CDR1* gene [18]. A previous study demonstrated that the minimum inhibitory concentration (MIC) of FLC was significantly decreased in *C. albicans* mutant with deletions in the *UPC2* gene (*upc2*∆/*upc2*∆) [12,19]. These findings suggested that inhibiting the transcriptional activity of Upc2 effectively reduces the tolerance of *C. albicans* to azoles and mitigates azole drug resistance. However, the mechanism by which *C. albicans*, in the absence of the *UPC2* gene, exhibits increased sensitivity to azoles remains unclear.

A recent study has demonstrated that Ellipticine and its analog phiKan 083 specifically target Ncp1, thereby disrupting the interaction between Erg11 and Ncp1. This disruption results in elevated levels of reactive oxygen species (ROS) in *C. albicans*, which subsequently induces protein oxidation and misfolding within the endoplasmic reticulum (ER), culminating in ER stress. The ER stress response facilitates the release of calcium ions (Ca^2+^) from the ER, induces mitochondrial Ca^2+^ accumulation and dysfunction, increases ROS production, and triggers apoptosis in *C. albicans* cells, thereby enhancing the antifungal efficacy of azoles [20]. These findings suggest that the disruption of the ergosterol biosynthetic pathway may contribute to ER stress in *C. albicans*. In this study, we utilized the *upc2*∆/*upc2*∆ mutant constructed isogenically in the *C. albicans* parental strain SN152, in which both copies of the *UPC2* gene were replaced with the auxotrophic markers *HIS1* and *ARG4* [12,21]. These auxotrophic markers do not affect azole susceptibility of *C. albicans* in nutrient-rich media such as an YPD medium, as used throughout this study [20,22]. We observed that the *C. albicans upc2*∆/*upc2*∆ mutant demonstrated increased sensitivity to ergosterol synthesis inhibitors and the calcium chelator ethylene glycol tetraacetic acid (EGTA), along with enhanced tolerance to calcium chloride (CaCl_2_), in comparison to the wild-type *C. albicans* strain SN152. These findings suggest that the deletion of the *UPC2* gene in *C. albicans* may disrupt Ca^2+^ homeostasis and dysfunction ER. Further investigations revealed that, under FLC treatment, the overall expression of genes involved in the ergosterol synthesis pathway and the unfolded protein response (UPR) pathway was significantly downregulated in the *upc2*∆/*upc2*∆ mutant compared to wild-type strain. Our results indicate that the absence of the *UPC2* gene impairs the UPR pathway by reducing the splicing of *HAC1* mRNA, thereby increasing the accumulation of unfolded proteins and ultimately enhancing the antifungal activity of azoles.

## 2. Materials and Methods

### 2.1. Strains, Primers, Agents, and Cultural Conditions

All strains, and primers used in this study are detailed in Supplementary Tables S1 and S2. The *C. albicans* strains were cultured in YPD medium, which consists of 2% dextrose (Sangon Biotech, Shanghai, China, A610219-0500), 1% yeast extract (Oxoid, Basingstoke, UK, LP0021B), and 2% peptone (Oxoid, LP0137), at 30 °C for liquid culture growth. For solid medium growth, YPD supplemented with 2% (*w*/*v*) agar was used, also at 30 °C unless otherwise specified. For the hyphal growth and biofilm formation assays, RPMI 1640 medium was utilized, comprising 10.4 g/L RPMI-1640 (Sigma-Aldrich, St. Louis, MO, USA), 3.45% MOPS, 0.2% NaHCO_3_, and 2% agar, adjusted to a pH of 7.0.

### 2.2. Antifungal Susceptibility Testing

The MIC assay was conducted following previously established protocols [23]. Briefly, compounds were subjected to a two-fold serial dilution across columns 2 to 11 of a 96-well plate, with 100 μL of liquid being removed from column 11. Subsequently, overnight cultures of *C. albicans* were adjusted to a cell density of 1 × 10^3^ cells/mL in YPD medium. A volume of 100 μL of this diluted *C. albicans* cell suspension was then added to each well of the plate. Column 1 served as the positive control, containing 100 μL of YPD and 100 μL of the *C. albicans* cell suspension, while column 12 acted as the blank control with 200 μL of YPD. The plates were incubated at 30 °C for 24 h without agitation to assess growth inhibition. Prior to the assay, the solutions were homogenized by pipetting, and absorbance was measured at 600 nm (OD_600_) using a spectrophotometer (Thermo Fisher Scientific, Multiskan SkyHigh, Waltham, MA, USA). The MIC was defined as the lowest concentration of the compound that resulted in a ≥50% reduction in fungal growth compared with the drug-free control well.

A dose-matrix titration assay was conducted as previously described to assess the synergistic effects of two drugs against *C. albicans* [19,24]. The drugs were serially diluted at a 2-fold ratio in separate 96-well plates, with one drug diluted along the columns and the other along the rows. Equal volumes of the solutions from the two plates were combined, resulting in wells containing various combinations of drug concentrations. Each well was inoculated with 100 µL of an overnight *C. albicans* culture, adjusted to a concentration of 1 × 10^3^ cells/mL, with a drug-free growth control included. Following incubation at 30 °C for 24 h, the optical density at 600 nm (OD_600_) was measured for each well using a spectrophotometer (Thermo Fisher Scientific, Multiskan SkyHigh, Waltham, MA, USA). The fractional inhibitory concentration index (FICI) was calculated based on the 24 h MIC results using the following formula: FICI = MIC_AB_/MIC_A_ + MIC_BA_/MIC_B_. Here, MIC_AB_ represents the MIC of drug A in the presence of drug B; MIC_A_ refers to the MIC of drug A alone. Similarly, MIC_BA_ represents the MIC of drug B in the presence of drug A; MIC_B_ refers to the MIC of drug B alone. The interaction between the drugs was classified as synergistic if the FICI was ≤0.5, additive if the FICI ranged from >0.5 to 1.0, indifferent if the FICI ranged from >1.0 to 4.0, and antagonistic if the FICI exceeded 4.0 [25].

### 2.3. Growth Inhibition Curve Assay

The growth inhibition curve assay was performed following the previously described methodology [26,27]. Briefly, overnight cultures of *C. albicans* colonies were inoculated into YPD medium and adjusted to a cell density of 1 × 10^3^ cells/mL. FLC was then serially diluted in a 96-well plate. Each well received 75 μL of the drug solution and 75 μL of the *C. albicans* culture. Wells containing 150 μL of YPD medium served as blank controls. The prepared plate was placed in the Infinite 200 PRO Multifunctional Microplate Reader (San Jose, CA, USA) for incubation and analysis. The incubation was conducted at 30 °C, with OD_600_ measured every 15 min over a 48 h period. Data analysis and graphical representations were generated using GraphPad Prism version 10.1.2.

### 2.4. Hypha Growth Assay

The hyphal growth assay was conducted as previously described [26,28]. *C. albicans* cells were cultured overnight in YPD medium at 30 °C with agitation until reaching the logarithmic growth phase. The OD_600_ of each culture (SN152 and *upc2*Δ/*upc2*Δ) was measured and adjusted to ensure equivalent cell densities. The cells were harvested by centrifugation and resuspended in RPMI 1640 medium. Subsequently, the suspension was diluted in RPMI 1640 to achieve a final concentration of 2 × 10^5^ cells/mL. A volume of 100 μL of this cell suspension, along with 100 μL of RPMI 1640 containing serial dilutions of FLC, was added to each well of a 96-well plate. Wells containing only RPMI 1640 served as blank controls. The plates were incubated at 37 °C for 4 h. Morphological differences between FLC-treated and untreated *C. albicans* cultures were observed and documented at 0, 1, 2, and 4 h using an inverted microscope (Motic, Hong Kong, China, AE2000).

### 2.5. Biofilm Formation Assay

The biofilm formation assay was conducted as previously described [26,28]. Following overnight culture in YPD medium, *C. albicans* SN152 and *upc2*Δ/*upc2*Δ cells were resuspended in RPMI 1640 medium and subsequently diluted to a concentration of 10^6^ cells/mL. A 96-well plate was utilized, with 100 μL of the cell cultures added to each well. The plates were incubated at 37 °C for 90 min to facilitate initial biofilm formation. Post-incubation, the culture medium was carefully removed, and each well was gently washed with sterile phosphate-buffered saline (PBS) to eliminate non-adherent cells. Fresh RPMI 1640 medium, with or without the addition of specific compounds, was then added to the wells, and the cells were cultured at 37 °C for an additional 24 h. This method necessitated the use of 2,3-bis-(2-methoxy-4-nitro-5-sulfophenyl)-2H-tetrazolium-5-carboxanilide (XTT), which undergoes metabolic reduction to produce a colorimetric, water-soluble formazan product in viable cells [29]. XTT was utilized in conjunction with phenazine methosulfate (PMS). The mixed solution was prepared immediately prior to use, with a ratio of XTT (0.5 mg/mL in DMSO) to PMS (0.32 mg/mL in sterile water) of 9:1, and subsequently stored in a dark environment. Following the completion of drug treatment, the medium was removed from the 96-well plate, and 100 μL of the XTT-PMS mixture was added to each well. The plates were then incubated at 37 °C in the dark for 30 min. The XTT-formazan results were analyzed using a microplate reader (Thermo Fisher, Multiskan SkyHigh) at a wavelength of 492 nm. Each sample was subjected to three replicates. Data were plotted using GraphPad Prism version 10.1.2, displaying the mean relative quantity ± standard deviation.

### 2.6. RNA Sequencing

The SN152 and *upc2*Δ/*upc2*Δ mutant cultures were initially grown overnight in YPD medium and subsequently incubated in YPD for an additional 8 h at 30 °C, with or without the addition of 4 μg/mL FLC. Following incubation, the biological samples were promptly frozen at −80 °C and dispatched for sequencing to BGI Genomics in Shenzhen, China. Differentially expressed genes (DEGs) under both untreated and FLC-treated conditions were identified based on the criteria of |log2 fold change (FC)| ≥ 1 and Q-value ≤ 0.05, and these DEGs were depicted using volcano plots.

### 2.7. Quantitative Real-Time PCR (qRT-PCR) Analysis of mRNA Expression Levels

*C. albicans* were cultured overnight and incubated for 8 h in the presence or absence of FLC (128 ng/mL). The extraction of total RNA were conducted according to the previous study [30]. Quantitative analysis of cDNA was carried out via real-time PCR using TB Green^®^ Premix Ex Taq™ II on a CFX96™ system (Bio-Rad, Hercules, CA, USA) with the following strategy: (1) 95 °C for 30 s; (2) 95 °C for 5 s, 50 °C for 30 s, and 72 °C for 30 s, for 40 cycles: (3) 95 °C for 5 s, 50 °C for 30 s, and 72 °C for 30 s. We used *ACT1* as the internal reference gene for normalization. Each sample was subjected to three technical replicates and the expression differences in the target gene were calculated using the 2^−ΔΔCt^ method [31].

### 2.8. Determination of Unfolded Protein Levels

The quantification of unfolded proteins within cells was conducted using flow cytometry [20,32]. To determine intracellular levels of unfolded proteins, overnight cultures of *C. albicans* were inoculated at a 1:100 dilution and cultivated in the presence or absence of FLC (128 ng/mL) for 4 h at 30 °C. A 5 μM concentration of tunicamycin was employed as a positive control for a 4 h exposure period. Following cultivation, the fungal suspension was resuspended in PBS. Subsequently, 500 μL of culture (10^7^ cells/mL) was treated with either 0.5 μL tetraphenylethene maleimide (TPE-MI) (50 mM) or an equivalent volume of DMSO, and the mixture was incubated in the dark at 37 °C with continuous agitation in a rotating incubator for 30 min. Post-incubation, the cells were washed with PBS and filtered through gauze for analysis using a BD FACSVerse flow cytometer (BD Biosciences, New York, NY, USA). The excitation and emission wavelengths for TPE-MI were set at 350 nm and 470 nm, respectively. Forward scatter (FSC) and side scatter (SSC) voltages were adjusted to 220 volts and 250 volts, respectively, to accurately encompass *C. albicans* cells while excluding cellular debris. The gating process was concluded upon reaching the stopping criterion of 10,000 events.

### 2.9. Detection of HAC1 mRNA Splicing

The determination of *HAC1* mRNA splicing was conducted as previously described [33]. Subsequently, 128 ng/mL of FLC was either added to the cultures or omitted, with 5 mM dithiothreitol (DTT) serving as the positive control. After a 2 h incubation period following compound addition, cells were collected via centrifugation. Total RNA was extracted using the Yeast RNA Extraction Kit (Zymo Research, Irvine, CA, USA, R1002). The quality and integrity of the RNA were assessed using the Agilent 2100 Bioanalyzer (Santa Clara, CA, USA) and formaldehyde gel electrophoresis. For the gel preparation, 0.28 g of agarose was dissolved in 20 mL of 1× MOPS buffer (2 mL of 10× MOPS buffer (200 mM MOPS, 50 mM sodium acetate, and 10 mM EDTA, with a pH of 7.0), and 18 mL of DEPC-treated water) (Sangon Biotech, Shanghai, China). Upon complete dissolution, 292 μL of formaldehyde and 2 μL of TS-GelRed (Tsingke Biotechnology, Beijing, China) were gently mixed into the solution, which was then poured onto the gel plate. Electrophoresis was conducted in 1× MOPS buffer at 80 V for 40 min, and RNA visualization was achieved using UV light. The integrity of the RNA was confirmed by the clear observation of the 28S and 18S bands, with the 28S band exhibiting twice the brightness of the 18S band. Subsequently, a *HAC1*-specific reverse primer was utilized to synthesize complementary DNA (cDNA), followed by polymerase chain reaction (PCR) amplification of the cDNA samples. Post-PCR, the relative abundance of *HAC1* transcripts was assessed through DNA electrophoresis on a 3% agarose gel.

To quantify *HAC1* splicing efficiency, a qRT-PCR approach was employed using two sets of primers: one set targeting the intron–exon junction to specifically amplify the unspliced *HAC1* (*HAC1^u^*) mRNA, and another set spanning the exon–exon junction to specifically amplify the spliced *HAC1* (*HAC1^s^*) mRNA. The remaining experimental procedures were performed identically to those of standard qRT-PCR. Each sample was subjected to three technical replicates and the expression differences in the target gene (*HAC1^u^* and *HAC1^s^*) were calculated using the 2^−ΔΔCt^ method [31]. The relative levels of *HAC1^s^* transcript were calculated by measuring the ratio of *HAC1^s^*/(*HAC1^u^* + *HAC1^s^*).

## 3. Results

### 3.1. Deletion of the UPC2 Gene in C. albicans Affects Physiological Processes That Are Dependent on the Calcineurin Signaling Pathway

**Figure 1:**
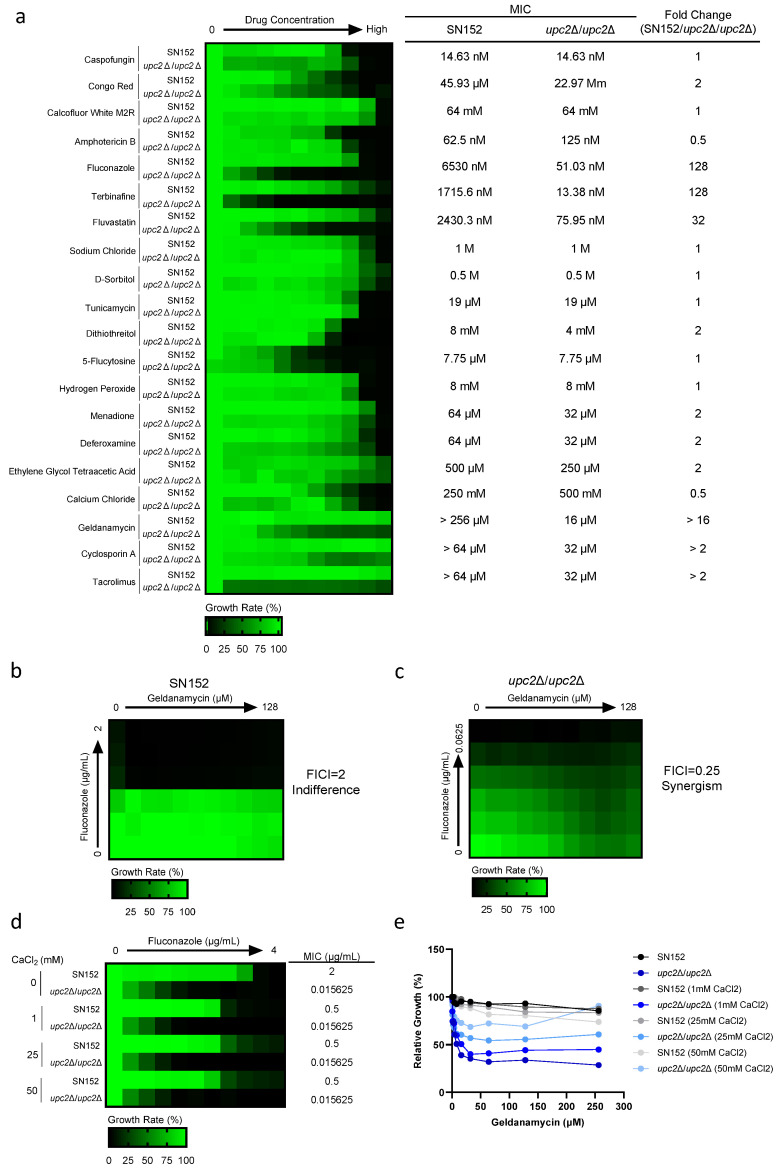
Deletion of the *UPC2* gene in *C. albicans* disrupts the Ca^2+^ homeostasis. (**a**) MIC assay to assess the susceptibility of the *C. albicans* wild-type strain SN152 and the *upc2*Δ/*upc2*Δ mutant to a panel of 20 compounds. (**b**,**c**) Dose matrix titration experiments of SN152 and *upc2*Δ/*upc2*Δ under different compound treatments. All strains were cultured in YPD medium at 30 °C for 24 h. (**d**,**e**) After supplementing with 1 mM, 25 mM, and 50 mM CaCl_2_, the MIC values of FLC and geldanamycin for SN152 and *upc2*Δ/*upc2*Δ were determined. All strains were cultured in YPD medium at 30 °C for 24 h.

We utilized a MIC assay to assess the susceptibility of the *C. albicans* wild-type strain SN152 and the *upc2*Δ/*upc2*Δ mutant to a panel of 20 compounds (Figure 1a). These compounds target various cellular processes, including the cell wall, cell membrane, osmotic pressure, endoplasmic reticulum stress, DNA damage, oxidative stress, iron homeostasis, and calcium homeostasis. The *upc2*Δ/*upc2*Δ mutant exhibited significantly heightened sensitivity to three inhibitors of ergosterol biosynthesis: FLC (an Erg11 inhibitor) [34], terbinafine (an Erg1 inhibitor) [35,36], and fluvastatin (an Hmg1 inhibitor) [35]. These results corroborate previous findings, indicating that the deletion of Upc2 markedly enhances the sensitivity of *C. albicans* to inhibitors of ergosterol synthesis.

The *upc2*Δ/*upc2*Δ mutant exhibits increased sensitivity to three drugs associated with Ca^2+^ homeostasis, including geldanamycin, an Hsp90 inhibitor [37,38], and the calcineurin inhibitors cyclosporine A and tacrolimus [39,40] (Figure 1a). We further performed a dose-matrix titration assay on SN152 and the *upc2*Δ/*upc2*Δ mutant to calculate the FICI between geldanamycin and FLC. The results indicated that geldanamycin and FLC demonstrated an indifferent interaction against SN152 (FICI = 2), whereas they exhibited a synergistic interaction against the *upc2*Δ/*upc2*Δ mutant (FICI = 0.25) (Figure 1b,c). These findings suggest that the deletion of the *UPC2* gene in *C. albicans* may disrupt Ca^2+^ homeostasis of the fungus.

To further substantiate this inference, we examined the sensitivity of the *upc2*Δ/*upc2*Δ mutant to the Ca^2+^ chelator EGTA and CaCl_2_. Our findings indicate that the *upc2*Δ/*upc2*Δ mutant demonstrated decreased sensitivity to EGTA and heightened sensitivity to CaCl_2_ (Figure 1a). Additionally, we administered varying concentrations of Ca^2+^ and subsequently determined the MIC of SN152 and *upc2*Δ/*upc2*Δ in the presence of FLC and geldanamycin (Figure 1d,e). Upon Ca^2+^ supplementation, the sensitivity of SN152 to FLC increased, whereas the sensitivity of *upc2*Δ/*upc2*Δ to FLC remained unchanged (Figure 1d). Notably, after the addition of 1 mM CaCl_2_, the MIC of *upc2*Δ/*upc2*Δ for geldanamycin increased from 16 μM to 32 μM, and with the addition of 25 mM and 50 mM CaCl_2_, the MIC exceeded 256 μM (Figure 1e). These findings suggest that the deletion of the *UPC2* gene in *C. albicans* impaired the calcium-dependent signaling pathway.

### 3.2. The Deletion of UPC2 Gene in C. albicans Affects the Expression of the ER Stress Related Genes

**Figure 2:**
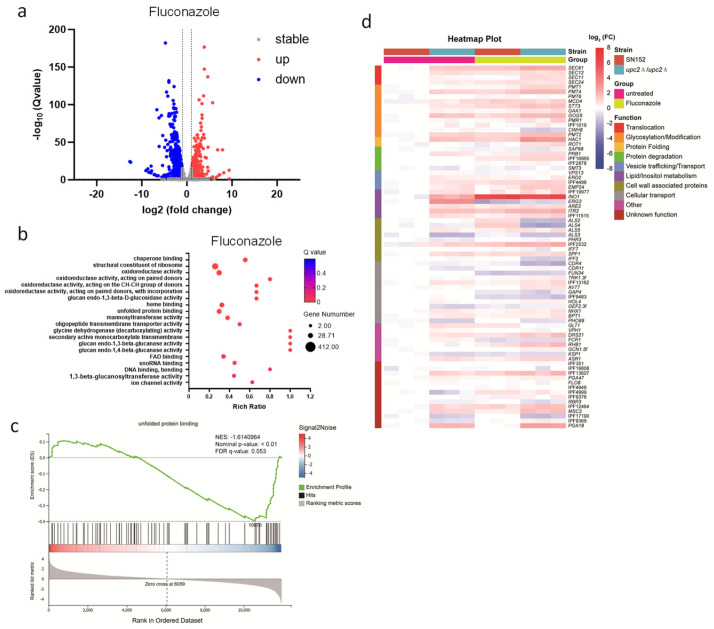
The deletion of *UPC2* gene in *C. albicans* affects the expression of the ER stress related genes. (**a**) When treated with 4 μg/mL FLC for 8 h, SN152 and *upc2*Δ/*upc2*Δ both showed differential genes. The differentially expressed genes were analyzed through RNA-seq. Each group of samples had 3 biological replicates, and the screening criteria for differential genes were |log2FC| ≥ 1 and Q ≤ 0.05. (**b**) Under the action of FLC, the DEGs of SN152 and *upc2*Δ/*upc2*Δ were enriched in the unfolded protein binding pathway. GO molecular function enrichment analysis of DEGs. (**c**) In GSEA of the DEGs, the gene set related to unfolded protein binding was downregulated. (**d**) TPM analysis of the DEGs.

The synthesis of ergosterol in *C. albicans* occurs within the ER, and increased intracellular Ca^2+^ concentrations may exacerbate ER stress in this organism [20]. Consequently, we propose the hypothesis that the deletion of the *UPC2* gene in *C. albicans* could induce ER stress. To evaluate this hypothesis, we utilized RNA sequencing (RNA-seq) technology to perform a transcriptomic analysis of *C. albicans* subjected to FLC treatment. Our findings indicate that, in comparison to the SN152 strain, the gene expression profile of the *upc2*Δ/*upc2*Δ mutant exhibited significant alterations. Under FLC treatment, 1817 DEGs were identified, comprising 867 upregulated and 950 downregulated genes (Figure 2a). Gene Ontology (GO) analysis of molecular function and biological processes revealed that the DEGs showed significant enrichment in aspects such as chaperone binding, structural constituent of ribosome, oxidoreductase activity, unfolded protein binding (Figure 2b). Gene Set Enrichment Analysis (GSEA) demonstrated that the Normalized Enrichment Scores (NES) for unfolded protein binding pathway was −1.61 (Figure 2c). To mitigate or manage ER stress, *C. albicans* cells utilize the UPR pathway, which plays a crucial role in restoring homeostasis and normal ER function [41]. The molecular function of unfolded protein binding is executed by ER chaperones, such as BiP, which detect and bind to misfolded proteins, thereby initiating the UPR and facilitating protein refolding during ER stress [42]. Research has demonstrated that in *C. albicans*, 76 genes are regulated by the transcription factor Hac1 and are involved in the UPR [43]. Notably, this curated UPR gene set is distinct from the gene set used for GSEA in Figure 2c. We therefore examined how deletion of the *UPC2* gene affects the expression of these well-characterized UPR genes in *C. albicans*, both in the presence and absence of FLC. Expression fold changes for each gene were quantified using transcripts per million (TPM) values in the SN152 and *upc2*Δ/*upc2*Δ strain. It is worth noting that when FLC is present, the absence of the *UPC2* gene leads to a decrease in the transcriptional levels of most of these UPR genes, such as *ALS2* (cell wall-associated protein gene), but the level of the *HAC1* gene, which is directly related to protein folding, increases relatively (Figure 2d). These findings suggested that the deletion of *UPC2* gene in *C. albicans* affects the expression of the ER stress related genes and the overall expression of the unfolded protein binding pathway is reduced in *upc2*Δ/*upc2*Δ strain.

### 3.3. The Deletion of UPC2 Gene Inhibits HAC1 mRNA Splicing in C. albicans

**Figure 3:**
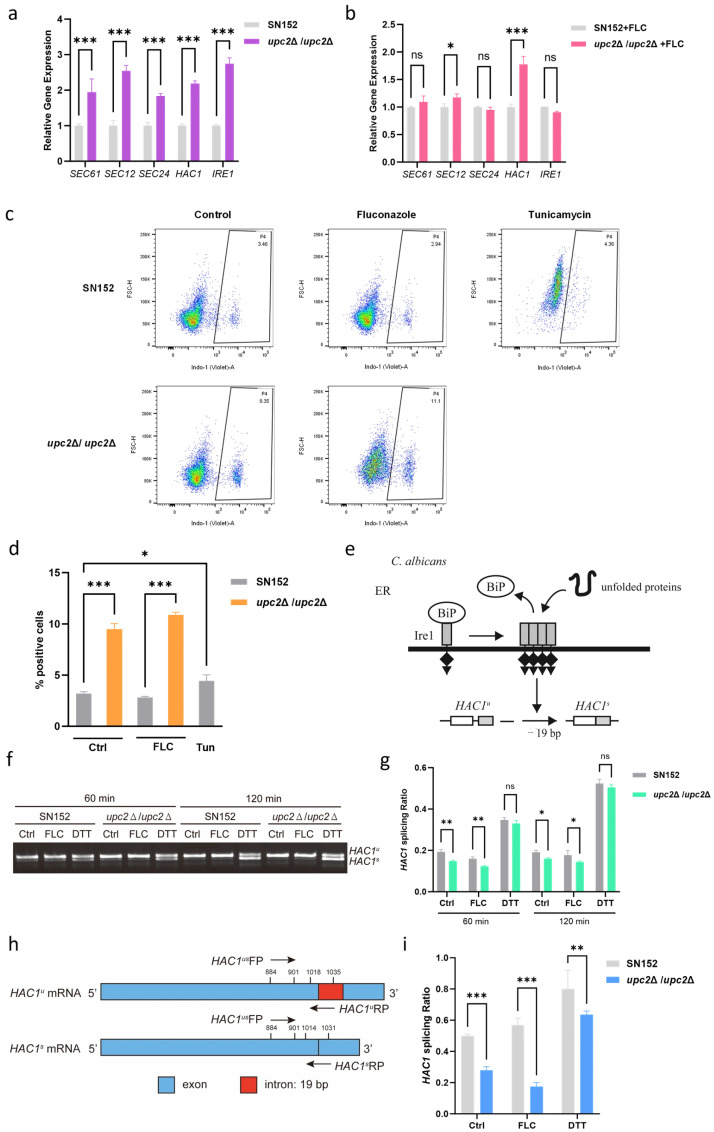
Deletion of the *UPC2* gene impairs *HAC1* mRNA splicing and potentiates unfolded protein accumulation in *C. albicans*. (**a**) The expression of genes in the unfolded protein binding pathway was analyzed using qRT-PCR in *C. albicans* SN152 and *upc2*Δ/*upc2*Δ strains. Data are expressed as mean ± SD for biological triplicates. Significance was determined by two-way ANOVA; *** *p* < 0.001. (**b**) *C. albicans* SN152 and *upc2*Δ/*upc2*Δ strains were treated with 128 ng/mL FLC for 8 h. Then, the expression of genes in the unfolded protein binding pathway was analyzed using qRT-PCR. Data are expressed as mean ± SD for biological triplicates. Significance was determined by two-way ANOVA; ns, no significant, * *p* < 0.05, *** *p* < 0.001. (**c**) The content of *upc2*Δ/*upc2*Δ unfolded proteins was higher than that of SN152 unfolded proteins. The content of unfolded proteins was determined using flow cytometry. 128 ng/mL FLC and 5 μM tunicamycin were treated for 4 h, with FSC at 220V, SSC at 250V, and Indo-1 at 360V. (**d**) Statistical analysis of the content of unfolded proteins. The data are presented as the mean ± SD for biological triplicates. Significance was determined by one-way ANOVA; * *p* < 0.05, *** *p* < 0.001. (**e**) Schematic diagram of translational control of *HAC1* mRNA in *C. albicans*. (**f**) The splicing rate of *HAC1* mRNA in SN152 and *upc2*Δ/*upc2*Δ was analyzed using RT-PCR. 128 ng/mL FLC and 5 mM DTT were used as positive controls. The Original Western blot images can be found in File S1. (**g**) Statistical analysis of the splicing rate of *HAC1* mRNA at 60 min and 120 min. The data were expressed as the average value ± standard deviation of 3 biological replicates. Significance was determined by two-way ANOVA; ns, no significant, * *p* < 0.05, ** *p* < 0.01. (**h**) Schematic diagram of qRT-PCR primers of *HAC1* mRNA. (**i**) Statistical analysis of the splicing ratio of *HAC1* mRNA at 120 min by qRT-PCR. 128 ng/mL FLC and 5 mM DTT were used. The data were expressed as the average value ± standard deviation of 3 biological replicates. Significance was determined by two-way ANOVA; ** *p* < 0.01, *** *p* < 0.001.

We selected five representative genes associated with the UPR pathway, specifically those involved in translocation and protein folding, designed primers for quantifying mRNA expression levels and validated their expression through quantitative PCR analysis. Under drug-free conditions, the *upc2*Δ/*upc2*Δ strain showed significant expression of unfolded protein-related genes, with relative expression levels exceeding 1.83-fold relative to the SN152 control (Figure 3a). Under FLC treatment, the *SEC12* and *HAC1* genes were notably overexpressed in the *upc2*Δ/*upc2*Δ strain, exhibiting relative expression levels of 1.17- and 1.78-fold compared to SN152, respectively (Figure 3b). To assess whether the absence of the *UPC2* gene influences the levels of unfolded proteins, we employed flow cytometry. Tetrathiophene maleimide (TPE-MI), a fluorescent dye with aggregation-induced emission properties capable of penetrating the cell membrane, was utilized to measure unfolded protein content [32,44]. As proteins unfold, cysteine residues previously buried in the folded core become exposed. This dye binds to the sulfhydryl groups of misfolded proteins, thereby emitting fluorescence [45]. Tunicamycin, an ER stress inducer known to inhibit N-glycosylation and promote the accumulation of misfolded proteins, was used as a positive control [46]. Our analysis revealed that the fluorescence levels in both the control group (DMSO) and the FLC-treated group were markedly elevated in the *upc2*Δ/*upc2*Δ strain compared to the SN152 strain (Figure 3c,d). This observation suggests that the deletion of the *UPC2* gene in the absence of drug treatment leads to an increase in the level of unfolded proteins, and this phenomenon is also observed in *C. albicans* under FLC treatment.

To mitigate ER stress and restore protein folding capacity, cells initiate the UPR as an adaptive mechanism [47]. The ER transmembrane receptor Ire1, possessing dual protein kinase and ribonuclease activities, is adept at detecting the excessive accumulation of unfolded proteins within the ER. It becomes activated through dimerization, oligomerization, and autophosphorylation across the ER membrane [48,49]. In response to ER stress, the activated Ire1 cleaves *HAC1* mRNA in yeast in a spliceosome-independent manner, thereby excising the intron sequences [50]. In the context of ER stress in *C. albicans*, Ire1 excises the intron fragment, and the resulting exons are ligated by tRNA ligase to produce *HAC1^s^* mRNA (Figure 3e). The precursor *HAC1* mRNA and its spliced variant are designated as *HAC1^u^* (unspliced) and *HAC1^s^* (spliced), respectively, distinguished by a 19-nucleotide fragment [33].

In this study, we assessed the splicing ratio of *HAC1* mRNA in SN152 and *upc2*Δ/*upc2*Δ strains under both untreated and FLC-treated conditions. Dithiothreitol (DTT) was utilized as a positive control. PCR products were resolved on a 3% agarose gel and visualized under UV light. The relative levels of the spliced *HAC1^s^* transcript were quantified by calculating the ratio of HAC1s to the sum of *HAC1^u^* and *HAC1^s^*. Our results indicated that, following 60 and 120 min of FLC treatment, the relative levels of *HAC1^s^* transcript in the *upc2*Δ/*upc2*Δ strain were significantly reduced compared to those in the SN152 strain (Figure 3f,g). Unlike the previously designed primers for *HAC1*, which target the regions 884–901 and 1017–1034 (Figure 3a,b), we further designed splice-specific qRT-PCR primers: one set spanning the exon-exon junction specific for *HAC1^s^*, and another set targeting the intron-exon junction specific for *HAC1^u^* (Figure 3h). The relative levels of *HAC1* splicing ratio was significantly lower in the *upc2*Δ/*upc2*Δ mutant than in the wild-type SN152 strain under untreated, FLC-treated, and DTT-treated conditions (Figure 3i). These findings suggest that the *UPC2* gene deletion impairs *HAC1* mRNA splicing in *C. albicans*. This impairment likely results in the accumulation of unfolded proteins, thereby increasing the *upc2*Δ/*upc2*Δ strain’s sensitivity to FLC.

### 3.4. The Deletion of UPC2 Gene Inhibits Hyphal Growth of C. albicans and Enhances FLC Against C. albicans Biofilm

**Figure 4:**
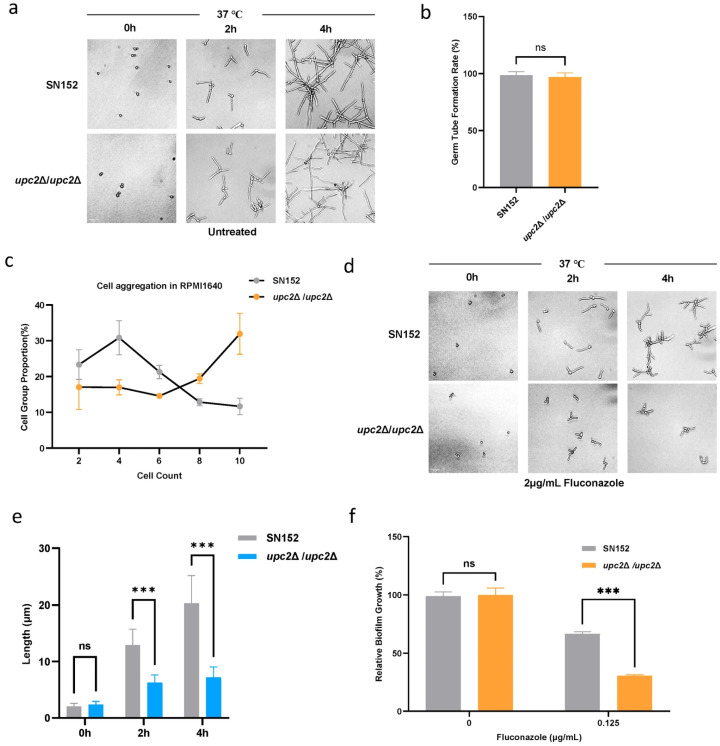
The deletion of *UPC2* gene inhibits hyphal growth and enhances the activity of FLC against biofilm of *C. albicans*. (**a**) The growth conditions of the hyphae at different times, with a scale of 20 μm. (**b**) Germ tube formation was assessed at 4 h post induction. At least 200 cells per sample were randomly selected and the percentage of cells with germ tubes was quantified. Data are presented as mean ± SD. (**c**) Following 4 h of aggregation, at least 60 cell clusters were scored per sample, and classified by the number of cells per aggregate: 1–2, 3–4, 5–6, 7–9, and ≥10 cells. The data are presented as the average value ± standard deviation of biological replicates. (**d**) Growth of SN152 and *upc2*Δ/*upc2*Δ hyphae at 0 h, 2 h, and 4 h, with a scale of 20 μm. SN152 and *upc2*Δ/*upc2*Δ were cultured in RPMI 1640 medium at 37 °C and 2 μg/mL FLC. (**e**) Length of SN152 and *upc2*Δ/*upc2*Δ hyphae at 0 h, 2 h, and 4 h, with data presented as the average value ± standard deviation of 30 biological replicates. Significance was determined by two-way ANOVA; *** *p* < 0.001. (**f**) The absence of *UPC2* significantly reduced the sensitivity of the biofilm formed during the growth of *C. albicans* to FLC. SN152 and *upc2*Δ/*upc2*Δ were cultured at 37 °C in RPMI 1640 medium. The data were expressed as the average value ± standard deviation of 3 biological replicates. Significance was determined by two-way ANOVA; ns, no significant, *** *p* < 0.001.

*C. albicans* exhibits a hyphal morphology that serves as a crucial virulence factor, facilitating invasion by breaching the mucosal barrier and penetrating the surfaces of biomedical materials such as medical silicone, thereby enabling the invasion of deep host tissues [51,52,53]. To investigate whether the deletion of *UPC2* gene influences hyphal growth, *C. albicans* SN152 and *upc2*Δ/*upc2*Δ strains was cultured in RPMI 1640 medium at 37 °C, and hyphal development was observed at 0, 2, and 4 h. In the absence of drug treatment (YPD, 30 °C), no significant difference in growth rate was observed between the SN152 and *upc2*Δ/*upc2*Δ yeast-form strains (Supplementary File S1). When transferred to hypha-inducing conditions (RPMI 1640, 37 °C), both SN152 and *upc2*Δ/*upc2*Δ strains were capable of hyphal growth, with no significant differences in hyphal morphology observed at the same time points (Figure 4a,b). Notably, we observed a tendency for the hyphae of the *upc2*Δ/*upc2*Δ strain to aggregate. After 4 h, the proportion of cell groups containing ten aggregated cells was higher in the *upc2*Δ/*upc2*Δ strain compared to the SN152 strain (Figure 4c).Upon treatment with FLC, *upc2*Δ/*upc2*Δ strain displayed a significantly shorter hyphal length compared to the SN152, indicating that the hyphal growth of the *upc2*Δ/*upc2*Δ strain was more significantly inhibited than that of SN152, and this difference in the growth lengths of the SN152 and *upc2*Δ/*upc2*Δ became more obvious over time (Figure 4d,e). These findings suggest that the absence of the *UPC2* gene impairs hyphal growth under FLC-induced stress.

Biofilms composed of yeast cells, pseudohyphae, and hyphae are initiated through the adsorption and adhesion of *C. albicans* yeast cells to a substrate. Once established, these biofilms exhibit a high tolerance to antifungal drugs, posing a substantial challenge in clinical treatment [54,55,56,57]. To elucidate the role of Upc2 in biofilm formation under FLC pressure, we employed the XTT reduction assay to assess the inhibitory effects of FLC on the biofilm formation of SN152 and *upc2*Δ/*upc2*Δ strains. XTT, a tetrazole salt, undergoes bioreduction to produce a water-soluble formazan, and is routinely utilized for the quantitative evaluation of *C. albicans* biofilms by measuring cellular activity [58,59]. Our findings indicate that at a concentration of 0.125 μg/mL FLC, the biofilm formation of the *upc2*Δ/*upc2*Δ mutant was significantly inhibited compared to that of SN152 (Figure 4f).

## 4. Discussion

In this study, we examined the mechanism by which the deletion of the *UPC2* gene enhances the antifungal efficacy of FLC against *C. albicans*. Our findings suggest that the *UPC2* gene deletion is associated with disrupted Ca^2+^ homeostasis and impaired activation of the UPR pathway by reducing *HAC1* mRNA splicing, which together contribute to the accumulation of unfolded proteins. This proteotoxic stress acts in concert with FLC to promote fungicidal effects on *C. albicans*. These results offer novel insights into the mechanisms underlying azole tolerance and identify potential therapeutic targets for addressing drug resistance in fungal infections.

Our preliminary chemical screening indicated that, beyond the inhibitors of ergosterol synthesis, the *upc2*Δ/*upc2*Δ mutant demonstrated markedly heightened sensitivity to compounds affecting Ca^2+^ homeostasis, notably the Hsp90 inhibitor geldanamycin. Through combination drug assays and Ca^2+^ supplementation experiments, we found that the deletion of the *UPC2* gene correlates with increased intracellular Ca^2+^ concentrations in *C. albicans*. This observation aligns with previous studies that have shown Hsp90 to physically interact with Upc2 and regulate its transcriptional activity [13,14]. Hsp90 serves as a major molecular chaperone with essential roles in both proteostasis maintenance and Ca^2+^ signaling, and regulates numerous drug resistance-related pathways in fungi [60,61,62,63]. In *C. albicans*, Hsp90 regulates echinocandin resistance via calcineurin, and depletion of Hsp90 mimics the azole sensitivity seen in calcineurin and protein kinase C mutants [38,64]. Our findings expand upon these observations by suggesting that Upc2 is functionally associated with Ca^2+^ homeostasis downstream of Hsp90. Given the dual roles of Hsp90 in Ca^2+^ signaling and proteostasis control, it is equally plausible that the geldanamycin sensitivity phenotype may also reflect perturbations in protein homeostasis and UPR function. Thus, disruption of the Hsp90-Upc2 regulatory axis may contribute to enhanced azole sensitivity through interconnected effects on Ca^2+^ homeostasis and proteostatic control.

The ER serves as the primary organelle responsible for protein folding, lipid synthesis, and Ca^2+^ storage within eukaryotic cells [65]. Disruptions in ER homeostasis, such as the accumulation of misfolded proteins or disturbances in Ca^2+^ signaling, activate the UPR pathway, an adaptive signaling pathway aimed at restoring proteostasis [41]. Our transcriptomic analysis demonstrated that, under FLC treatment, genes associated with the UPR pathway were significantly downregulated in the *upc2*Δ/*upc2*Δ mutant relative to the wild-type strain. Notably, we observed that the absence of the *UPC2* gene is associated with compromised the splicing of *HAC1* mRNA, a crucial step for the *UPR* pathway activation. In yeast, the ER transmembrane kinase/ribonuclease Ire1 initiates UPR signaling through the unconventional splicing of *HAC1* mRNA, resulting in the production of the active transcription factor Hac1, which subsequently induces the expression of UPR target genes [48,50]. The diminished splicing efficiency of *HAC1* mRNA in the *upc2*Δ/*upc2*Δ mutant is accompanied by impaired the UPR pathway activation, with a corresponding the accumulation of unfolded proteins. This accumulation of unfolded proteins creates a proteotoxic stress that synergizes with FLC to inhibit fungal growth.

Calcium signaling has emerged as a promising target for combination antifungal therapy [66]. Several studies have shown that targeting Ca^2+^ signaling pathways, such as using Ca^2+^ channel blockers or calcineurin inhibitors, can significantly enhance the efficacy of azoles against *C. albicans*, particularly in biofilm-associated infections [5,63]. Our finding that the *UPC2* gene deletion disrupts Ca^2+^ homeostasis is consistent with this body of work and provides another link between ergosterol biosynthesis, Ca^2+^ signaling, and azole tolerance. The observation that supplementation with exogenous Ca^2+^ increased the MIC of geldanamycin in the *upc2*Δ/*upc2*Δ mutant further supports the conclusion that the antifungal effect of geldanamycin involves disruption of Ca^2+^ homeostasis. This suggests that combining Upc2 inhibitors with Ca^2+^ signaling blockers could be an effective strategy for enhancing azole efficacy.

An important implication of this study is that targeting the UPR pathway offers a promising therapeutic strategy to overcome azole tolerance. Traditional strategies to combat azole resistance have largely focused on inhibiting ergosterol biosynthesis [6,12]. However, our findings indicate that interfering with the UPR pathway, specifically through the splicing of *HAC1* mRNA, can significantly enhance the antifungal efficacy of FLC. This notion is supported by recent work showing that disrupting the interaction between Erg11 and Ncp1 enhances azole efficacy by inducing ER stress and increasing ROS production [20]. Together, these studies support the idea that impairing the ability of *C. albicans* to resolve proteotoxic stress represents a viable strategy for converting fungistatic azoles into fungicidal agents.

Several questions remain to be addressed in future studies. Specifically, we did not conduct complementation experiments, such as reintroducing the *UPC2* gene or over- expressing the epitopic *HAC1*^s^ in the *upc2*Δ/*upc2*Δ mutant. Such assays are critical for definitively validating the direct causal relationship between the *UPC2* gene deletion, impaired *HAC1* splicing, and the accumulation of misfolded proteins. In the absence of these functional validations, our current findings support a robust correlation among the above-mentioned molecular events. However, the absolute causality cannot be fully confirmed, and potential indirect effects triggered by the *UPC2* gene deletion cannot be entirely ruled out. Restricted by objective experimental conditions, these rescue experiments were not performed in the present work. Future studies will focus on completing such functional validation to further solidify the regulatory mechanism identified in this study. Second, the precise molecular mechanism by which Upc2 regulates *HAC1* mRNA splicing remains unclear. It is possible that Upc2 directly or indirectly regulates the expression of components of the UPR pathway, or that the effect is secondary to changes in ergosterol content and membrane fluidity. Third, it would be important to determine whether inhibiting Upc2 or the UPR pathway is effective against azole-resistant clinical isolates of *C. albicans* and other pathogenic *Candida* species such as *C. auris*, where Upc2 overexpression is frequently associated with drug resistance [17]. Fourth, in vivo studies are needed to evaluate the efficacy of combining Upc2 inhibitors with FLC in animal models of candidiasis. Finally, it would be interesting to explore whether there are synergistic interactions between UPR inhibitors and other classes of antifungal agents beyond azoles.

## 5. Conclusions

In conclusion, our study indicates that loss of the *UPC2* gene in *C. albicans* is associated with enhanced antifungal effect of FLC. This enhanced effect is not only related to the downregulation of genes in the ergosterol synthesis pathway, but is likely also associated with UPR inhibition, particularly the reduction in *HAC1* mRNA splicing and the increase in unfolded protein accumulation. These findings reveal a previously unrecognized role for Upc2 in modulating proteostasis and Ca^2+^ homeostasis, extending beyond its canonical function in regulating ergosterol biosynthesis. Our results support the idea that development of small molecules targeting the UPR pathway may represent an effective strategy to potentiate the efficacy of FLC and counter azole resistance in *C. albicans*.

## Data Availability

The RNA-Seq data from this study has been submitted to the NCBI under the BioProject ID: PRJNA1288166 (http://www.ncbi.nlm.nih.gov/bioproject/1288166, accessed on 23 April 2026).

## Supplementary Materials

The following supporting information can be downloaded at: https://www.mdpi.com/article/10.3390/pathogens15060629/s1, File S1. Original Western blot images of Figure 3f; Table S1: Strains were used in this study; Table S2: Primers were used in this study.

## Abbreviations

The following abbreviations are used in this manuscript:

Hmg1	3-hydroxy-3-methylglutaryl-CoA reductase
FLC	fluconazole
NLS	nuclear localization signal
DBD	DNA-binding domain
LBD	ligand-binding domain
SREs	sterol response elements
ERGs	ergosterol biosynthesis genes
MIC	minimum inhibitory concentration
ROS	reactive oxygen species
ER	endoplasmic reticulum
Ca^2+^	calcium ions
EGTA	ethylene glycol tetraacetic acid
UPR	unfolded protein response
