# Supplementary material for: Disruption of the UPC2 Gene Enhances Fluconazole Antifungal Activity by Inhibiting HAC1 mRNA Splicing in Candida albicans
Source: Pathogens. 2026 Jun 12;15(6):629. doi: 10.3390/pathogens15060629 (PMC13304746; doi:10.3390/pathogens15060629)
Supplement: Supplementary file 1 [file pathogens-15-00629-s001.zip › Supplementary Tables.pdf]

Table S1. Strains were used in this study.

No.	Alias	Name	Genotype	Source
1	*C. albicans*	SC5314	Wild type	Eukaryot Cell. 2005 Feb;4(2):298-309.
2	*C. albicans*	SN152	his1 /his1 arg4/arg4 leu2 /leu2	Eukaryot Cell. 2005 Feb;4(2):298-309.
3	*C. albicans*	*upc2Δ/upc2Δ*	upc2::HIS1 /upc2::ARG4 leu2 /leu2	mBio. 2023 Feb 28;14(1):e0263922.
4	*S. cerevisiae*	BY4741	MATa his3Δ1 leu2Δ0 met15Δ0 ura3Δ0	Mar Drugs. 2019 Jan 14;17(1):54.

Table S2. Primers were used in this study.

No.	Primer name	Primer sequence(5' to 3')
1	ACT1-S	TTGATTTGGCTGGTAGAG
2	ACT1-AS	ATGGCAGAAGATTGAGAA
3	ERG10-S	TGAAGCCGCCAGAAATCC
4	ERG10-AS	TGACCCAAGGCAACAGCA
5	ERG13-S	TCTAAATCCGTCAAGTCG
6	ERG13-AS	ATCACCAGCAACAACAAT
7	HMG1-S	GACAAGATCCAGCTCAAA
8	HMG1-AS	GGACCACGAACACCTAAT
9	ERG12-S	TTGACAGGTGCTGGAGGT
10	ERG12-AS	GTGGCATCGTCTAAATCTTC
11	ERG8-S	GCTCAGAAACACCCAAAT
12	ERG8-AS	AATCGCAACAGTCAAAGG
13	IDI1-S	TGACGAATCTTCCAAATG
14	IDI1-AS	GTCCAAGTTATCCCACCA
15	ERG20-S	AACATTTCCGTCAAGATCCTTACTA
16	ERG20-AS	TGCTAATGCCACGGGTAA
17	ERG9-S	ATCCCTTTGTTGCGTGAA
18	ERG9-AS	CCATACCATTACCCATTT
19	ERG1-S	AAAACCTCCTTCTGCTGC
20	ERG1-AS	CCAACTGTCATACCACCC
21	ERG7-S	GACATCAGGGACGAAATA
22	ERG7-AS	TTGGCAAAGTAAGTAAGC
23	ERG11-S	TTTGGTGGTGGTAGACAT
24	ERG11-AS	CTGCTGGTTCAGTAGGTAAA
25	ERG24-S	CCGCTCAGCAAATAAACA
26	ERG24-AS	CCAATCAACCAGTCACCC
27	ERG25-S	TACTTGGCATTATTGGTTTC
28	ERG25-AS	GCATCAACGGCTTGGAAT
29	ERG26-S	TTAGTCTACACCTCCTCAG
30	ERG26-AS	TCATTATCGTTGGCTTTC
31	ERG27-S	CCCAACTAAAGTAAACAAG
32	ERG27-AS	CAATCTATGCCTCCGTAT
33	ERG28-S	CTCCATTATCAGCAAGAA
34	ERG28-AS	CAAATACATCCAAACCAA
35	ERG6-S	TTGTGGTGTAGGTGGTCC
36	ERG6-AS	CTTCAATGGCATAAACAG
37	ERG2-S	TAATAATGCTGGTGGTGC
38	ERG2-AS	GGTAAATAAACTTCGGGAAT
39	ERG3-S	ATGAATGTACTGGTGGTT
40	ERG3-AS	GCAATGGGAATAATAATGG
41	ERG5-S	AGATACCGTCCACCAGTC
42	ERG5-AS	CATAATTCTTACCCAAACAC
43	ERG4-S	GGGGTTCCATTCAGTTAC
44	ERG4-AS	AAATCGGAATAAGGCAAG
45	UPC2-S	CGGAATCAACTACTCAAC
46	UPC2-AS	CACCTAACCCAGAAAGAT
47	NCP1-S	CATTGTCGCTACTTATGG
48	NCP1-AS	GCAAATCTTTCACCTCCT
49	HAC1-S	ACCCATCACCACTTTCAT
50	HAC1-AS	AGACTGATTGCTGCTAAA
51	SEC61-S	TGGCTTCCAATAGAGGTA
52	SEC61-AS	CATAGACGGTGGCTTGAC
53	SEC12-S	TGGTGGAGGAGAAGGTAA

54	SEC12-AS	CAACTAATTTGGCGGTGT
55	SEC24-S	GCCTTCAGACCACAGAGC
56	SEC24-AS	TCCAGCACCTTCCTCATC
57	IRE1-S	CGGAAACTGATACTGAAA
58	IRE1-AS	CCTCCACGACTACCTCTT
59	BY4741HAC1specificRP	CCCACCAACAGCGATAATAACGAG
60	BY4741HAC1FP	CGCAATCGAACTTGGCTATCCCTACC
61	BY4741HAC1RP	CCCACCAACAGCGATAATAACGAG
62	SN152HAC1FP	AGACGCTTTTTGGAATTACCCATCACCA
63	SN152HAC1RP	TCAAAGTCCAACTGAAATG
64	SN152HAC1specificprimer	GCGTGGTCATATGTTTAACCATC
65	HAC1usFP	ACCAGAACAACAAGAACA
66	HAC1uRP	TAGACTGATTGCTGCTAA
67	HAC1sRP	ATTGGCTCTGCTAAATCA
