# Supplementary figures and images for: Disruption of the UPC2 Gene Enhances Fluconazole Antifungal Activity by Inhibiting HAC1 mRNA Splicing in Candida albicans
Source: Pathogens. 2026 Jun 12;15(6):629. doi: 10.3390/pathogens15060629 (PMC13304746; doi:10.3390/pathogens15060629)

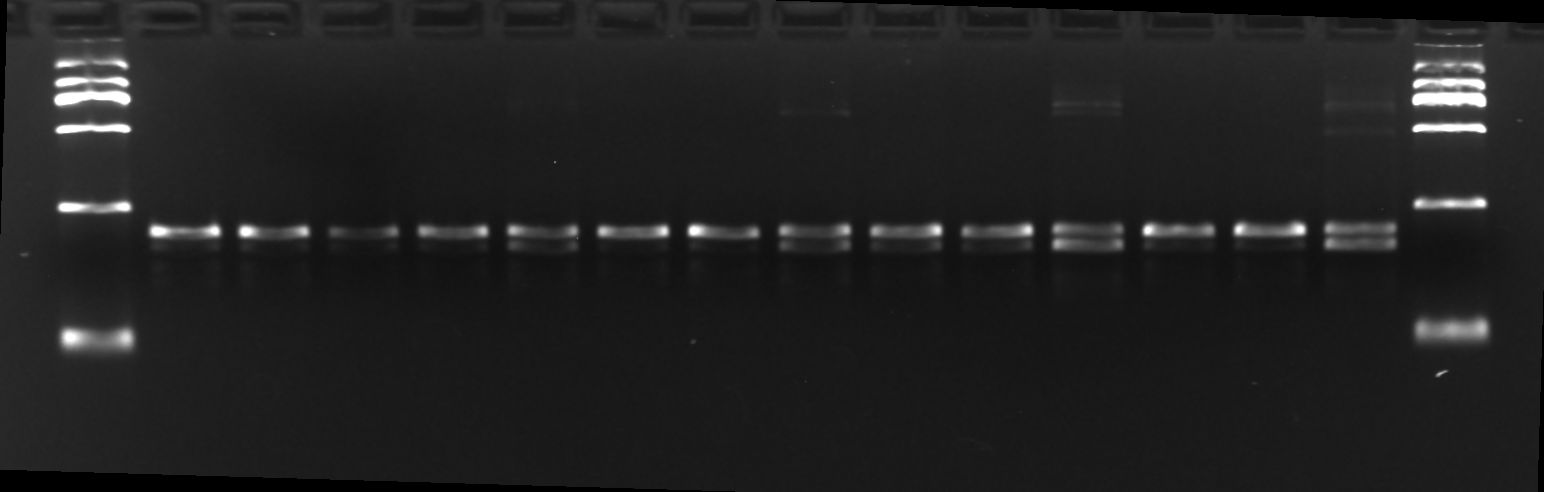

Supplement: Supplementary file 1 [file pathogens-15-00629-s001.zip › File S1. Original Western blot images of Figure 3f/2025-10-14 UV-channel1crop斜.tif]

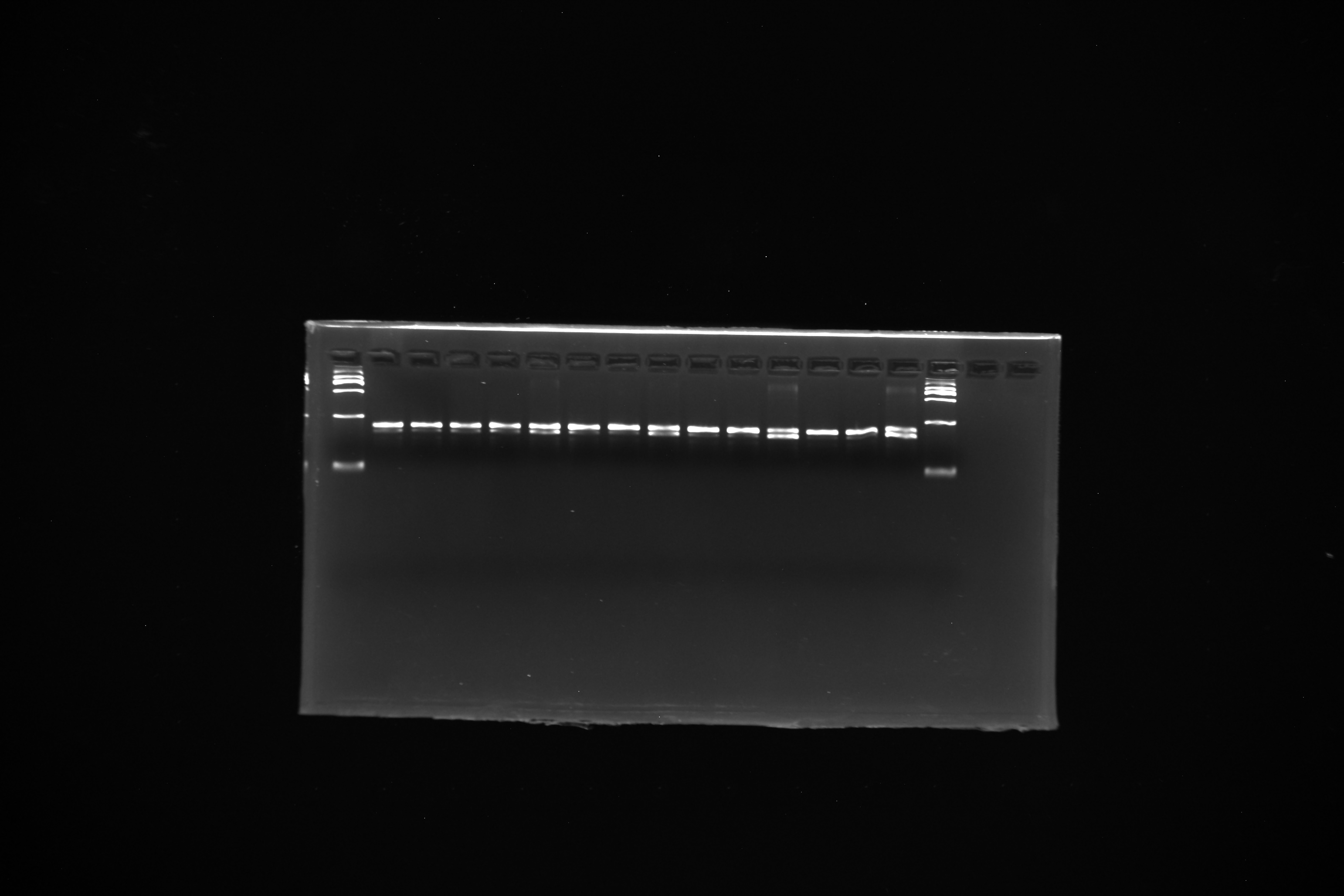

Supplement: Supplementary file 1 [file pathogens-15-00629-s001.zip › File S1. Original Western blot images of Figure 3f/666-2025-10-17 14-49-32-UV-channel2.tif]

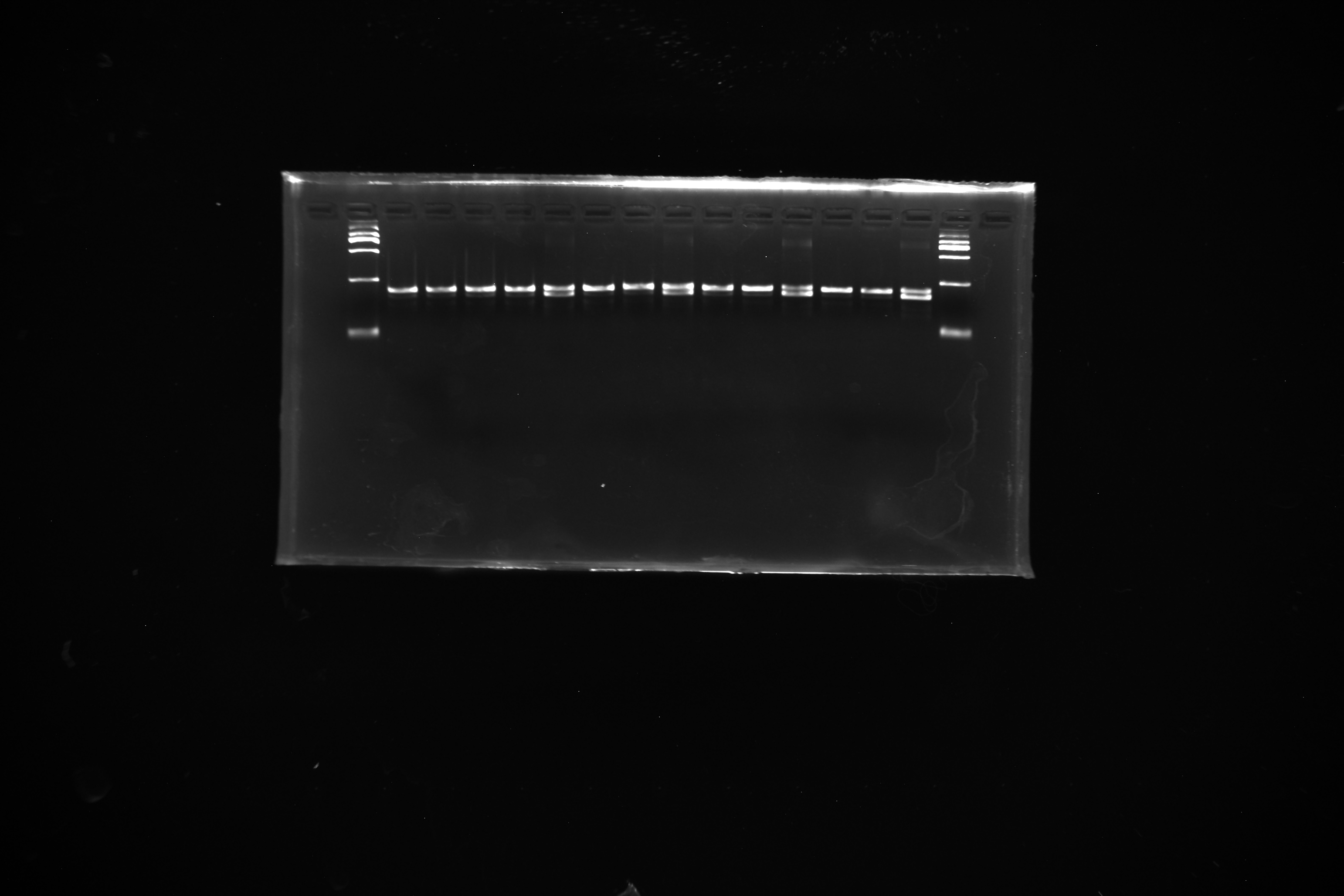

Supplement: Supplementary file 1 [file pathogens-15-00629-s001.zip › File S1. Original Western blot images of Figure 3f/666-2025-11-02 10-10-59-UV-channel2.tif]
